# Detailed quantum mechanical, molecular docking, QSAR prediction, photovoltaic light harvesting efficiency analysis of benzil and its halogenated analogues

**DOI:** 10.1016/j.heliyon.2019.e02825

**Published:** 2019-11-14

**Authors:** Y. Shyma Mary, Y. Sheena Mary, K.S. Resmi, Veena S. Kumar, Renjith Thomas, B. Sureshkumar

**Affiliations:** a Department of Physics, Fatima Mata National College (Autonomous), Kollam, Kerala, India; b Department of Physics, SN College, Kollam, Kerala, India; c Department of Chemistry, St Berchmans College (Autonomous), Changanassery, Kerala, India; d Department of Chemistry, SN College, Kollam, Kerala, India

**Keywords:** Organic chemistry, Theoretical chemistry, Pharmaceutical chemistry, DFT, Docking, NLO, MEP, DSSC

## Abstract

The structural, spectroscopic various physico-chemical and biological characteristics of the organic molecule benzil (BZL) and derivatives, 1,2-bis(4-methylphneyl)-1,2-ethanedione (DMB), 4,4′-difluorobenzil (DFB), 4,4′-dichlorobenzil (DCB) and 4,4′-dibromobenzil (DBB) have been studied by various computational methods. The experimental and scaled simulated Raman and IR spectra were compared and found close agreement. Assignments of important peaks are also presented. Detailed information pertaining to the local and global reactivity and other properties like electrophilic and nucleophilic characteristics were analysed. The hyperactive pressure was measured in terms of polarizability and corresponding biological properties were validated to identity reactive sites. Prediction of Activity Spectral Studies (PASS) predicts the biological activity of the compounds and it is found that the candidate molecules can be used as feruloyl esterase inhibitor, bisphosphoglycerate phosphatase inhibitor and Prolylaminopeptidase inhibitor. The crystals structures of those receptors are taken from the protein data bank and docking studies indicates stable complex with the receptors and candidate molecules. Light harvesting efficiency, followed by photovoltaic modelling shows that DMB is the best compound to be used in the DSSC to get the best output.

## 1 Introduction

Benzil, an yellow coloured solid is usually synthesised by the oxidation of benzoin, an aromatic 1,2-diketone which is widely used as an important organic intermediate and organic raw material for the manufacture of different types of drugs, insecticides and also used for curing free radicals during the synthesis of various polymers [1]. Bakkiyaraj et al. [2] reported the spectral features of benzyldioxime. Elmaci et al. [3] have studied the synthesis and DFT studies of benzilmonohydrazone based Schiff bases. Benzil single crystal growth and characterization is reported in literature [4]. Many benzyl derivatives have shown excellent nonlinear optical properties and dielectric properties [5]. Diphenylethane-1,2-dione analogues were reported to inhibit mammalian carboxylesterases, which is an enzyme used during the metabolism of xenobiotics [6, 7]. The synthesis, spectroscopic characterization, thermal study and biological evaluation of nickel and copper complexes with tetradentate ligand which was derived from benzil and 5-amino-1,3,4-thiodiazole-2-thiol was reported by Chandra et al. [8]. Benzil has been identified as a potent selective inhibitor of carboxylesterases [9]. Harada et al. [10] reported the comparison of benzil and trifluoromethyl ketone (TFK)-mediated carboxylesterase inhibition using classical and 3D-quantitative structure activity relationship analysis. Subarkhan et al. [11] reported the synthesis of a series of binuclear ruthenium(II) arene benzil bis(benzoylhydrazone) complexes and screened for their cytotoxicity activities. Evangelisti and Caminati [12] reported the rotational spectrum of 3,5-difluorobenzyl alcohol by pulsed jet Fourier transform microwave spectroscopy. The single crystal study of 4,4′-dibromobenzil with room temperature phosphorescence as well as controllable elastic and plastic bending are reported recently [13]. In spite its pharmaceutical importance, after review the literatures, there was no recent work found to explore all the fundamental and customized properties of title compounds in focus with the pharmaceutical properties. It is necessary to explicit the structural, biological, vibrational and unknown physico-chemical properties to understanding the direct and indirect applications of the title compounds. The spectral analysis of benzil (BZL) and similar derivatives, 1,2-bis(4-methylphneyl)-1,2-ethanedione (DMB), 4,4′-difluorobenzil (DFB), 4,4′-dichlorobenzil (DCB) and 4,4′-dibromobrnzil (DBB) are performed and compared with theoretical values. Various other quantum mechanical properties like stability, frontier molecular orbital properties, electrostatic potential, first order hyperpolarizability analysis to predict the non linear activity, light harvesting efficiency analysis, electronic spectra simulation, biological activity prediction followed by molecular docking and quantitative structure activity relationship to predict the biological properties as a function of observed descriptors were also performed in high levels of theory.

## 2 Computational calculations

This manuscript reports various computational studies on the target molecules. Gaussian 09W software was used for all calculations [14] along with Gauss View [15] for visualisations. All calculations were performed using the common B3LYP function using cc-pVDZ basis sets. Geometry optimised is certainly a energy minimum conformation. Potential energy scan (PES) was performed to study the conformational preference. The IR and Raman spectra were also simulated using the same level of theory for all the molecules after making sure that no imaginary frequencies are present in the optimised structure. The generated data was scaled using a scaling factor of 0.9613 [16] and compared with the experimental spectra [17]. FMO and MEP studies were also performed in the same level of theory. TD-DFT simulations were used to study the electronic spectra of the compounds and suitability of compound to be used in dye sensitised solar cells (DSSC). For that CAM-B3LYP functional was used as it could extend the time dependent electronic transitions well than the conventional B3LYP functional. The same basis set was used and the calculations were performed in a solvent cage of methanol using the IEPCM model.

## 3 Results and discussion

### 3.1 Conformational analysis

**Fig. 1:**
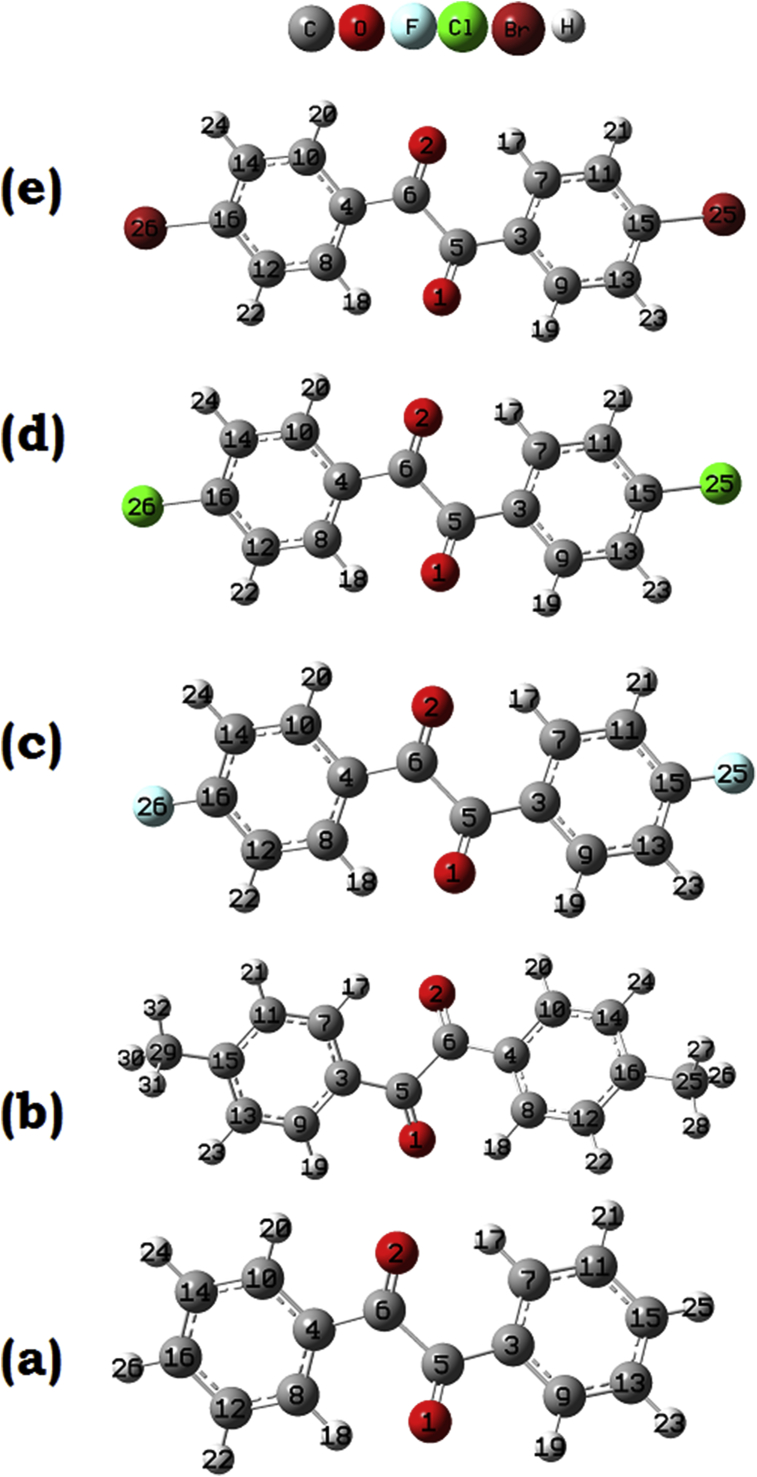
Optimized geometry of (a) BZL (b) DMB (c) DFB (d) DCB (e) DBB.

**Fig. 2:**
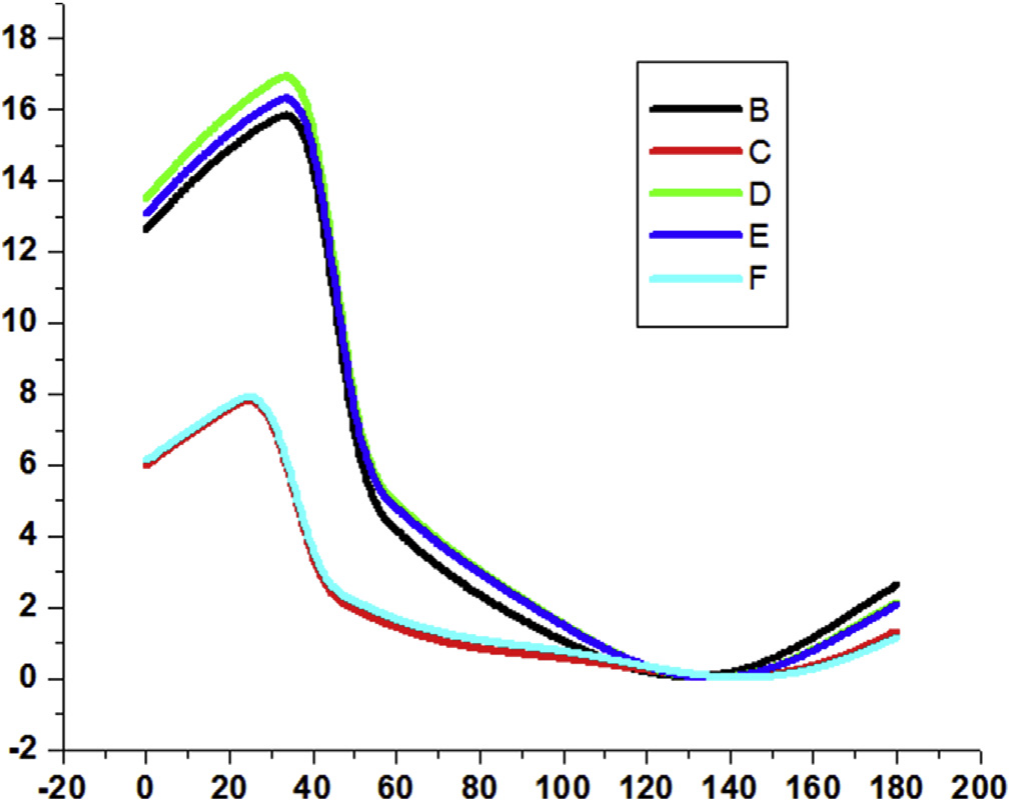
PES about the dihedral angle C4–C6–C5–C3 [(B) BZL (C) DMB (D) DFB (E) DCB (F) DBB].

Conformation studies provide insight into the preferential orientation of the molecule. Fully relaxed potential energy scan is performed on all the five benzil derivatives on C4–C6–C5–C3 bonds (Fig. 1). PES scan data are given in the Fig. 2 and for the unsubstituted benzil, the global maximum is at about 40° and global minima were at about 130° for all the compounds. The conformational energy is found to be of 16.3175 kJ/mol. The energy barrier is maximum for DFB, followed by DCB, BZL, DBB and DMB. In the case of the dimethyl derivative, the global maximum is at 30°, minimum is at 140° and the conformational energy is of 8.2860 kJ/mol. In the case of DFB, the conformational energy is at 17.4132 kJ/mol where the maxima and minima are at 40 and 140° respectively. For DCB, the conformational energy is at 16.4048 kJ/mol where the maxima and minima are at 40 and 140° respectively. But in the case of DBB, the conformational energy is at 8.3755 kJ/mol where the maxima and minima are at 30 and 130° respectively. The highest barrier of DFB is due to the high electron negativity of the fluorine atom, which causes greater polarisation and repulsion as they are nearer to each other. At the global minima, the substituents are the farthest away from the phenyl ring of the adjacent carbonyl providing more stability by reducing steric repulsions.

### 3.2 Electronic spectra and photovoltaic modelling

The electronic spectra of the compounds were simulated using the Time dependent density functional theory formalism (TD-DFT) using long range correction incorporated CAM-B3LYP functional and cc-pVDZ basis set in methanol solvent cage using IEPCM solvation model [18]. In the case of BZL molecule, there is only one significant electronic transition at 276.30 nm with oscillating strength (f) 0.608. These transitions are basically due to HOMO-3 to LUMO (13%), HOMO-2 to LUMO (67%) and HOMO to LUMO (15%). The HOMO of the molecule is located near the carbonyl groups and the three carbon atoms near the carbonyl carbon of the both rings, but the LUMO is delocalised over the entire molecule. The HOMO-2 is localised over atoms. When electrons are excited from HOMO-2 to LUMO (67%), there will be extensive electron delocalisation which stabilises the molecule.

DMB is a dimethyl derivative of the diketone BZL, with methyl groups in the para position to ketone in either rings. simulation of electronic spectra indicates a major transition at 286.99 nm with oscillator strength 0.8355, This transition is due to HOMO-1 to LUMO (78%) and HOMO to LUMO+1 (17%). The HOMO is located near the carbonyl group, HOMO-1 in the aromatic rings and LUMO+1 is concentrated in the carbonyls. The major transition is from HOMO-1 to LUMO, indicating the electronic transition from the aromatic rings to the antibonding orbitals of the carbonyl.

DFB is a diflouro derivative of the diketone with F atoms in the para position of the both rings. The major UV absorption is at 271.83 nm, with oscillator strength 0.7299, which is due to HOMO-1 to LUMO (83%) and HOMO to LUMO+1 (13%). HOMO is located in the entire molecule including the F atoms, while HOMO-1 was located over the aromatic rings with localised electrons over the F atoms and carbonyl groups and in LUMO, the orbitals are delocalised over the carbonyl groups. The major transition is between the delocalised electrons in the phenyl ring towards the antibonding orbitals over the carbonyls.

In DCB, two chlorine atoms are presents in the molecule, each in the para position to the carbonyl. Simulated UV spectra show one significant peak of oscillator strength 0.9581 at 277.26 nm, which is due to the HOMO-1 to LUMO (81%) and HOMO to LUMO+1 (15%). In this molecule, the HOMO are delocalized over the Cl and the three carbon atoms of the phenyl ring near the Cl atoms, and the electrons are localized over the carbonyl group. In HOMO-1, the orbitals are delocalized over the halogens, carbonyls and the carbon atoms attached to the Cl in aromatic systems and in the LUMO, the delocalized orbitals are prominent over the aromatic carbons to which the carbonyls are attached. The electronic transition is between the HOMO-1 to LUMO, which is dominant.

In the case of the dibromo derivative, a prominent absorption at 279.96 nm is found with oscillator strength 1.036. This transition is due to HOMO-1 to LUMO (79%) and HOMO to LUMO+1 (15%). The HOMO of this molecule was found to be delocalised over the carbonyl carbons and localized over the Br and adjacent carbon atoms. HOMO-1 was delocalized over the entire molecule except the bromine atoms, and LUMO had vacant antibonding orbitals over the carbonyl system. There electronic transition is found to be between the delocalized orbitals in the aromatic system to the unoccupied antibonding orbitals at the carbonyl.

Light harvesting efficiency (LHE) is the ability of the molecule to act as the photo sensitizer. It is a direct function of the oscillator strength of the molecule given by the equation LHE = 1-10^−f^, when f is the oscillator strength [19, 20, 21].

Data provided in Table 1 shows that LHE is maximum for DBB with value 0.9079. It shows that the compound can absorb light and can transfer 90.70 % of the energy from light is transferred to the acceptor system, here the semiconducting material. Least LHE is observed for BZL. Vertical electron excitation energy is defined as the energy required for the excitation of the electron, and is obtained from the λ(max) value. Using the vertical excitation energy, the electrons in ground state of the dye move to the excited state and transfer that excess energy to the electrons in the valence band of the semiconducting material in the solar cell, here TiO_2_. The band gap of TiO_2_ is 4.00 eV and if the excitation energy is more that the band gap, the compound can effectively promotes the electron from the valence band to the conduction band. This data can be used to calculate the free energy of the electron ejection, ΔG_injection_. More the negative value of the free energy, more spontaneous will be the DSSC. Photovoltaic modelling shows that DMB is the best compound to be used in the DSSC to get the best output. The value of BZL is almost same as that of DMB.

**Table 1:** Photovoltaic modeling of the compounds of the series.

	BZL	DMB	DFB	DCB	DBB
f	0.608	0.8355	0.7299	0.9581	1.036
LHE	0.753396	0.853951	0.813748	0.889871	0.9079
λ(max) (nm)	276.39	286.99	271.83	277.26	279.96
E(0,0) (eV)	4.486414	4.320708	4.561675	4.472336	4.429204
E(HOMO) (eV)	-6.59604	-6.42352	-6.79496	-6.82217	-6.84639
E(LUMO) (eV)	-2.62862	-2.46399	-2.7293	-1.75405	-2.84359
Eg (eV)	3.967422	3.959531	4.065655	5.068123	4.002797
Edye (eV)	6.596043	6.423523	6.794959	6.82217	6.846388
E*dye (eV)	2.109629	2.102815	2.233284	2.349834	2.417184
E(CB) (eV)	-4	-4	-4	-4	-4
ΔGinject (eV)	-1.89037	-1.89718	-1.76672	-1.65017	-1.58282

### 3.3 Frontier molecular orbital analysis

**Fig. 3:**
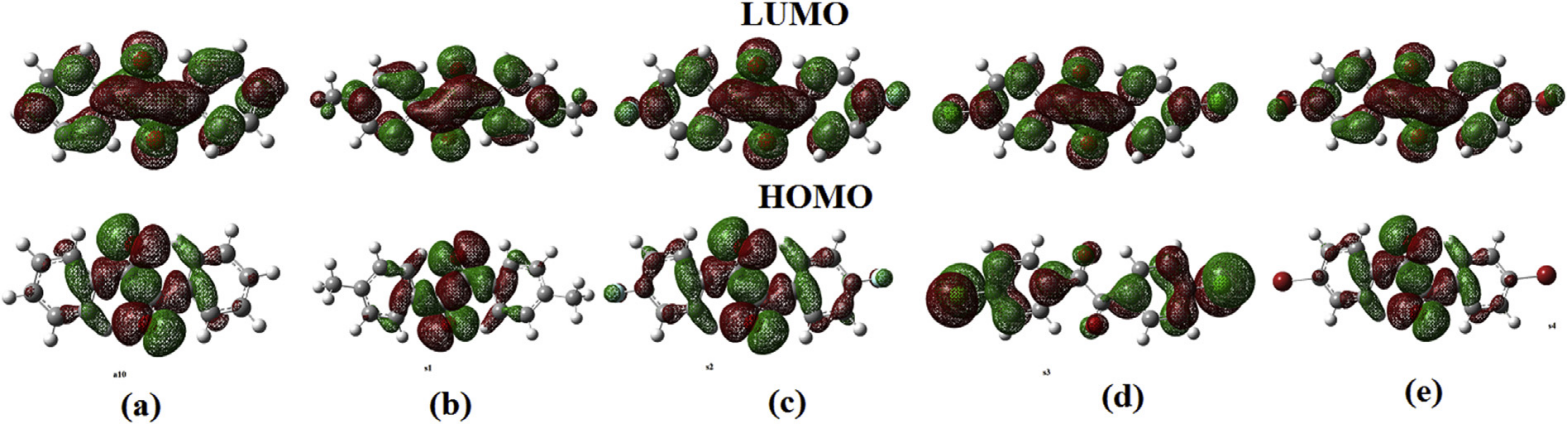
HOMO-LUMO plots of (a) BZL (b) DMB (c) DFB (d) DCB (e) DBB.

HOMO and LUMO are very important orbitals that decide the reactivity of the molecules [22, 23]. The main cause of chemical reactivity is the gap between the HOMO and LUMO of a particular molecule [23]. The present study, the molecular orbitals are modelled from the optimised geometry obtained using B3LYP/cc-pVDZ simulation, which is represented in the Fig. 3. The energy of HOMO and LUMO can be used for the calculation of various other physico-chemical properties like electron affinity (EA), which is negative of energy of LUMO, Ionisation potential (IP) of the molecule, which is again the negative of HOMO. Electronegativity χ is the average of the sum of electron affinity and ionisation potential given by the equation (IP + EA)/2. The important parameter chemical potential, which predicts the spontaneity of the chemical reactivity is the negative of electron negativity, represented by μ = -(IP + EA)/2. Chemical hardness η is half the difference between IP and EA as represented as (IP-EA)/2. Chemical softness is the reciprocal of hardness (S = 1/η) and electrophilicity index, which gives the electrophilic property of the molecule is ω = μ^2^/2η. Hardness is the measure of resistance of the system to change the electron cloud, while softness is the reciprocal of harness, which is the measure of ability to undergo electron density distribution. Softness varies in the order DCB > BZL > DBB > DMB > DCB. Chemical potential is the available energy in the system to perform useful work [24, 25, 26]. Table 2 gives the data of the chemical descriptors of the five molecules. Ionisation potential is minimum for DCB and maximum for the unsubstituted BZL which changes in the order DCB < DFB < DMB < DBB < BZL. On halogenation ionisation potential decreases. The electronegativity values also show the similar trend. But the electron affinity is more for BZL, but minimum for DFB, which indicates that DFB is having fewer tendencies to gain electrons compared to that of the other substituted benzyl derivatives. Chemical potential values, which are a very useful indicator of reactivity of the compounds, indicated that the least value is for DCB and high value is for the unsubstituted BZL form. This shows that the BZL is more reactive when compared to the analogues and the substitution with any type (+M or –M) will definitely decreases the reactivity of the compound and increases the stability of the compound. The energy gap decreases in the order DFB (2.979) > DMB (2.809) > DBB (2.789) > BZL (2.683) > DCB (2.466) and the reported values are 3.51 eV for benzildioxime [2] and in the range 3.39–4.05 eV for a series of benzilmonohydrazone derivatives [3].

**Table 2:** Chemical descriptors.

Compounds	IP = -E_HOMO_	EA = -E_LUMO_	χ=(IP + EA)/2	μ = -(IP + EA)/2	η =(IP-EA)/2	S = 1/η	ω = μ^2^/2η
BZL	8.748	6.065	7.407	-7.407	1.342	0.745	20.441
DMB	8.689	5.880	7.285	-7.285	1.405	0.712	18.887
DFB	8.681	5.702	7.192	-7.192	1.490	0.671	17.357
DCB	8.174	5.708	6.941	-6.941	1.233	0.811	19.537
DBB	8.694	5.905	7.300	-7.300	1.395	0.716	19.100

### 3.4 Nonlinear optical studies

Nonlinear optical properties are very important properties and they find applications in the discovery of novel organic electronic materials like OLED, organic transistors, organic semiconductors etc. [27]. First order hyperpolarizability data are presented in the Table 3 and they are obtained as a result of Raman spectral calculations. The values are in the order-DBB (8.295×10^−30^esu) >DCB (6.704×10^−30^esu) >DMB (3.989×10^−30^esu)> DFB (3.716×10^−30^esu) > BZL (1.927×10^−30^esu) which are 64, 52, 31, 29 and 15 times that of urea while the second order values are -3.807×10^−37^, -14.386×10^−37^, -13.540×10^−37^, -20.195×10^−37^ and -25.090×10^−37^ for BZL, DMB, DFB, DCB and DFB [28]. Higher values of first order hyperpolarizabilities indicate that they can act as a better nonlinear optical material than the standard material urea, in a relative scale. Thus the reported co-crystals can be considered as a crystalline system, which is having potential to be developed as NLO materials and also in organic electronics. For benzildioxime, the dipole moment, polarizability and first order hyperpolarizability values are respectively, 5.00 Debye, 1.5×10^−23^esu and 0.571×10^−30^esu [2].

**Table 3:** NLO properties.

Compound	μ	α ×10^−23^ esu	β ×10^−30^ esu	γ ×10^−37^ esu
BZL	2.4212	2.275	1.927	-3.807
DMB	2.7404	2.757	3.989	-14.386
DFB	1.4873	2.305	3.716	-13.540
DCB	1.3013	2.745	6.704	-20.195
DBB	1.3607	2.966	8.295	-25.090

### 3.5 Molecular electrostatic potential (MESP)

**Fig. 4:**
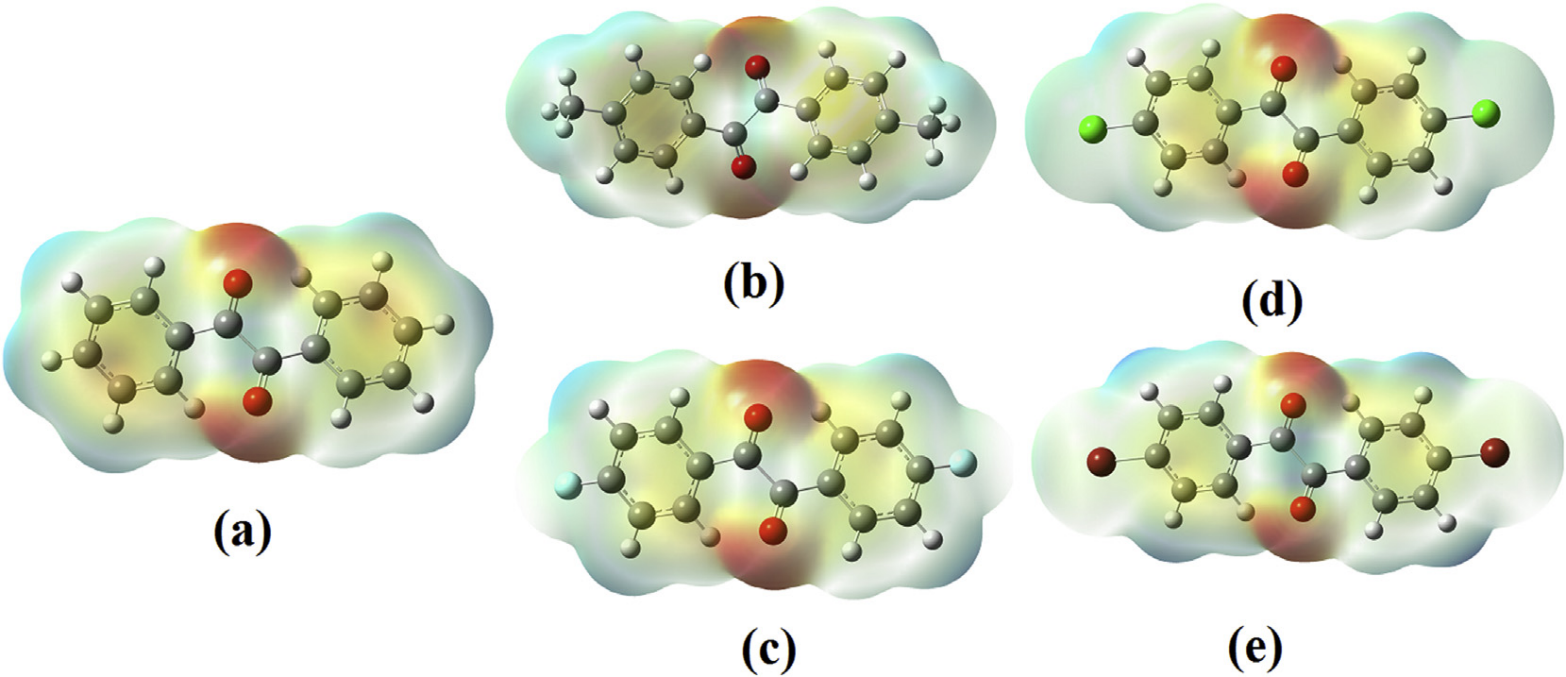
MEP plots of (a) BZL (b) DMB (c) DFB (d) DCB (e) DBB.

Molecular electrostatic potential (MESP) map is the three dimensional presentation of the charge distribution in the molecules, which allows to visualise the charge points in the molecule and shape of the potential surface, which helps to predict a variety of chemical properties [29]. GaussView sets the calculated electrostatic energy into an electron density model derived from the Schrodinger equation with the help of a colour spectrum in the order order, red > orange > yellow > green > blue. Red colour indicates electrophilically active region and blue colour indicates nucleophilically active region. In the present molecule (See Fig. 4), oxygen atom at the carbonyls are deep red in colour indicating that it is the maximum electrophilic region, where the nucleophiles attack followed the phenyl rings. Deep blue regions are present around the hydrogen atoms, showing that they are the highest nucleophilic region in the molecule susceptible to the attack of the electrophile [30, 31].

### 3.6 Molecular docking studies

PASS (Prediction of Activity Spectra) [32] gives activities, Feruloyl esterase inhibitor, Bisphosphoglycerate phosphatase inhibitor and Prolylaminopeptidase inhibitor (activity values 0.934, 0.931 and 0.930) and the corresponding receptors are, 3WMT, 2H4Z and 2EEP are used for docking. PatchDock Server is used for docking purpose [33, 34, 35, 36] and the algorithm of Patchdock has three major steps: molecular shape representation, surface patch matching and filtering and scoring [37, 38, 39]. Feruloylesterases (FAEs) (3WMT) are carboxyl esterases which enhance the hydrolysis of ester bonds between ferulic acid and polysaccharides present in the plant cell wall [40]. Bisphosphoglyceratemutase (2H4Z) is an enzyme which is specific on erythrocyte which synthesize 2,3-diphosphoglycerate, which is an allosteric effect of hemoglobin. This ezyme deficiency causes an increased affinity of oxygen by the cells [41]. Dipeptidyl aminopeptidase-4 (2EEP) is a present in the cell surface as a peptidase class of enzyme which is actively engaged in the regulation of blood glucose levels [42, 43].

When the compounds are docked with 3WMT, the interactions are: The residues of amino acid Glu336 form H-bond with carbonyl group whereas Ala561 has a π-alkyl interaction with phenyl ring; Arg356 gives π-sigma, amide π-stacked and π-alkyl interaction with phenyl ring of BZL. The residues of the amino acid Lys426 form H-bond with carbonyl and Met415 have π-sulfur interaction with phenyl ring. Cys271, Leu274, Pro451 shows alkyl interaction with CH3 while Ile291, Arg522, Pro451 exhibits π-alkyl interaction for DMB. Pro451, Gln270, Gln289 form halogen interaction with fluorine and Pro451, Arg522 exhibits π-alkyl interaction with phenyl ring. Asp284, Ile291, Met415 shows π-anion, π-sigma, π-sulfur interactions respectively with phenyl for DFB. Amino acid Gln270 forms halogen interaction and His516 shows π-alkyl interaction with chlorine atom. Asn523 exhibits H-bond and Cys271, Pro451, Cys517, Arg522, Ile291 shows π-alkyl interaction with phenyl ring for DCB. Amino acid Gln289 forms H-bond and Cys271 has a halogen interaction with Br but Asp 284 shows π-anion interaction with the phenyl ring. Cys271, Cys288, Pro451 forms alkyl interaction with Br atom and Arg522, Ile291, Pro451, Cys517 shows π-alkyl interaction with DBB.

When the title compounds are docked with 2H4Z the interactions are:The residues of amino acid Leu69, Lys29, Met35 show π-alkyl interaction and His65 having π-π-stacked interaction with BZL. The residues of the amino acid His65, form H-bond with carbonyl and Glu72 has π-anion interaction with phenyl ring. Met35, Leu29, His65 shows alkyl interaction with CH3 while Met35 exhibits π-alkyl with DMB. **T**he residues of the amino acid Met35 forms π-sulfur and π-alkyl interaction with phenyl ring. Glu72 exhibits π-anion interaction and Leu69 shows two π-alkyl interactions with DFB. Lys29 having π-cation, π-alkyl and alkyl interaction with phenyl ring while Glu72, His65 forms π-anion, π-π-T shaped interaction respectively with phenyl ring. Met35, Leu69 shows π-alkyl interaction with chlorine whereas His65, Trp68 forms π-alkyl interaction with chlorine of DCB. His65, Trp68 forms π-π T shaped halogen interaction with phenyl ring. Lys29, Met35, Trp68 forms alkyl interaction with Br atom and Lys29, Met35, Leu69 shows π-alkyl interaction with phenyl ring of DBB.

When the title compounds are docked with 2EEP the interactions are: The residues of amino acid Ala294, Pro440, Pro520, Lys521 forms π-alkyl interaction and Lys521 having π-π-stacked interaction with phenyl rings of BZL. Amino acid Glu336 forms π-lone pair and Arg356 has amide-π-stacked interaction with phenyl ring. Leu338having alkyl interaction with methyl group whereas Arg356, Ala561 shows π-alkyl interaction with phenyl ring of DMB. The residues of the amino acid Leu338 form H-bond whereas Asp331, Glu336 shows halogen interaction with fluorine. Leu338, Ala561, Arg356 shows π-alkyl interaction and Glu72 exhibits π-anion interaction with phenyl ring of DFB. The residues of the amino acids Glu336, Gln565 having π-Donor H-bond and Asp331 forms π-anion interaction with phenyl ring. His339 shows π-alkyl interactions with chlorine atom whereas Ala561, Arg356 gives π-alkyl interaction with chlorine of DCB. The residue of the amino acid His339 forms H-bond and π-alkyl interaction with Br whereas Glu336 has a H-bond with carbonyl group. Leu338 shows π-sigma interaction with phenyl ring and alkyl interaction with Br atom. Arg356 forms π-sigma, amide π-stacked, π-alkyl interaction with phenyl rings while Val335 shows a π-alkyl interaction with phenyl ring of DBB.

**Fig. 5:**
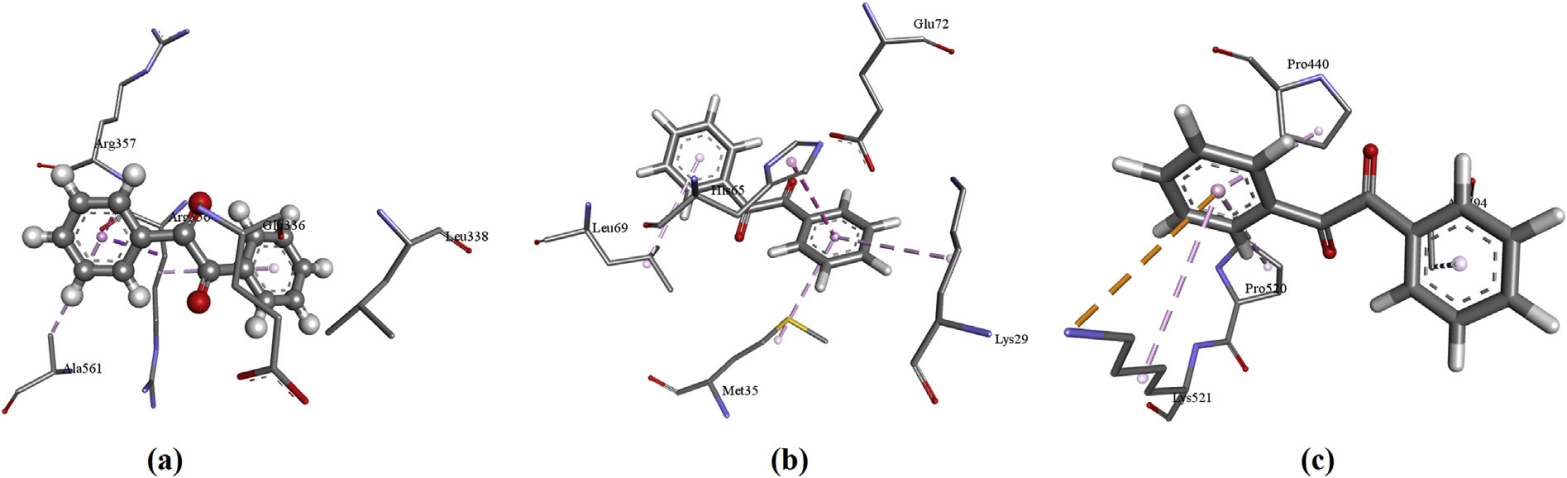
The interactive plot of docked ligand BZL with (a) 3WMT (b) 2H4Z (c) 2EEP

**Fig. 6:**
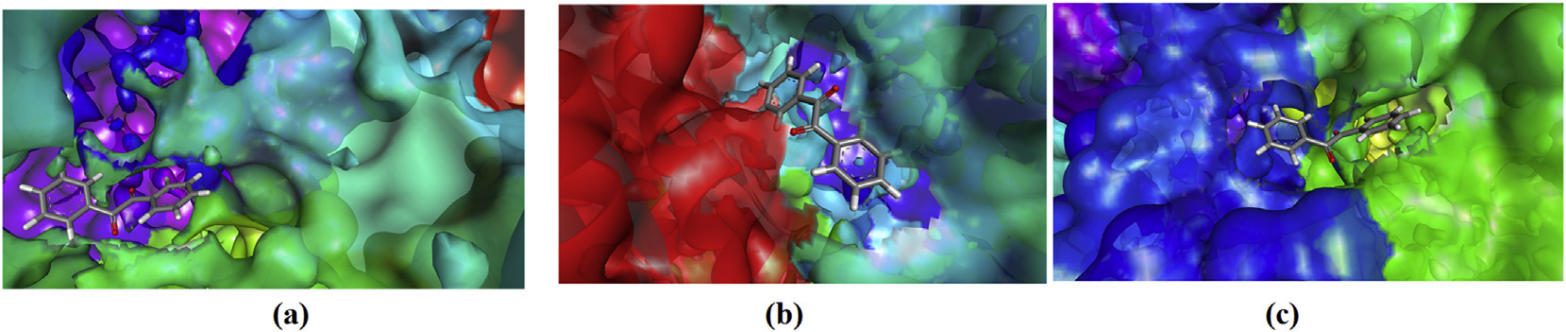
The docked ligand BZL with a) 3WMT (b) 2H4Z (c) 2EEP at the active sites of proteins.

The plot of docked ligand, BZL with receptors and at active sites is shown in Figs. 5 and 6. From Table 4, atomic contact energy value of BZL is high in comparison with that of other compounds for the feruloyl esterase inhibitor (3WMT) and prolylaminopeptidase inhibitor (2EEP) while DMB has high atomic contact energy value for bisphosphoglycerate phosphatase inhibitor (2H4Z). Gobal energy is high for DBB for feruloyl esterase inhibitor (3WMT) and DCB for bisphosphoglycerate phosphatase inhibitor (2H4Z) and prolylaminopeptidase inhibitor (2EEP).

**Table 4:** The top ten conformation of the complex candidate of ligands with proteins of energy values generated by Patch Dock Server.

No.	Global Energy	Attractive Vdw	Repulsive Vdw	Atomic Contact Energy
**Table 4.1**				
**3WMT-BZL**				
1	-23.12	-9.89	4.69	-8.89
2	-22.40	-9.37	3.32	-7.28
3	-22.39	-9.33	1.97	-6.41
4	-21.62	-9.92	3.44	-6.90
5	-21.33	-8.85	3.53	-7.27
6	-21.21	-9.44	2.25	-6.61
7	-20.80	-9.32	1.99	-7.46
8	-19.79	-10.17	3.96	-6.08
9	-18.92	-8.30	4.00	-6.42
10	-18.73	-13.07	6.32	-3.36
**3WMT-DMB**				
1	-24.78	-10.07	1.31	-7.12
2	-24.54	-10.40	5.70	-9.13
3	-24.37	-11.14	3.77	-7.44
4	-24.10	-10.37	1.78	-6.51
5	-24.03	-12.91	5.20	-8.43
6	-23.32	-11.90	5.83	-7.53
7	-23.20	-9.63	1.00	-6.50
8	-22.87	-9.18	1.90	-7.11
9	-22.65	-11.20	2.45	-5.43
10	-22.40	-13.66	14.98	-9.73
**3WMT-DFB**				
1	-25.78	-13.65	5.34	-6.05
2	-23.18	-10.64	1.29	-5.33
3	-22.44	-10.90	1.30	-4.80
4	-21.90	-12.53	2.69	-3.31
5	-19.70	-12.95	4.31	-5.21
6	-19.40	-11.26	3.22	-4.23
7	-19.19	-9.89	2.94	-5.38
8	-18.18	-9.30	3.84	-5.60
9	-17.20	-11.55	5.01	-3.63
10	-17.08	-9.03	4.74	-4.81
**3WMT-DCB**				
1	-25.24	-11.33	3.80	-8.31
2	-23.64	-13.42	5.13	-7.17
3	-23.61	-11.87	2.71	-6.66
4	-23.00	-10.29	3.92	-6.87
5	-22.06	-10.67	4.99	-7.58
6	-21.63	-10.08	2.45	-5.91
7	-21.53	-13.29	10.29	-6.19
8	-21.04	-10.61	0.89	-5.94
9	-19.80	-9.87	4.32	-6.61
10	-19.47	-9.76	2.88	-4.81
**3WMT-DBB**				
1	-26.46	-11.30	1.12	-6.81
2	-25.22	-10.66	1.28	-6.91
3	-21.31	-9.77	4.16	-6.80
4	-20.62	-13.87	2.51	-3.93
5	-20.27	-9.74	1.11	-5.84
6	-19.88	-8.86	2.12	-5.46
7	-19.85	-13.10	14.49	-8.47
8	-19.53	-8.49	3.25	-6.23
9	-19.39	-9.99	3.26	-5.56
10	-19.26	-10.86	2.66	-4.50
**Table 4.2**				
**2H4Z-BZL**				
1	-27.52	-12.80	3.27	-8.97
2	-22.05	-9.77	3.30	-6.83
3	-20.75	-13.94	2.85	-2.92
4	-20.26	-10.66	5.96	-8.11
5	-19.95	-9.22	2.02	-5.30
6	-19.25	-11.99	0.61	-5.16
7	-18.23	-8.30	0.93	-5.54
8	-17.52	-10.49	2.56	-2.70
9	-17.39	-9.36	2.64	-5.59
10	-15.93	-8.29	4.67	-5.19
**2H4Z-DMB**				
1	-32.38	-16.91	9.27	-11.11
2	-27.40	-13.48	3.67	-8.78
3	-27.20	-13.09	3.24	-8.75
4	-21.92	-11.62	4.16	-5.09
5	-21.64	-14.85	2.80	-2.93
6	-21.55	-9.89	4.28	-6.83
7	-19.46	-9.76	4.49	-6.84
8	-19.21	-9.37	2.83	-6.02
9	-18.82	-10.18	5.51	-6.19
10	-18.80	-9.17	1.63	-4.77
**2H4Z-DFB**				
1	-21.76	-12.66	2.37	-5.57
2	-18.13	-13.01	3.33	-3.39
3	-15.95	-8.81	2.97	-4.31
4	-15.59	-10.01	4.89	-4.62
5	-15.16	-7.62	2.25	-5.32
6	-14.79	-10.57	3.89	-1.96
7	-14.44	-8.81	1.99	-2.30
8	-14.39	-9.84	4.14	-4.78
9	-13.51	-11.55	7.07	-1.73
10	-13.41	-11.71	2.13	0.59
**2H4Z-DCB**				
1	-25.92	-14.18	8.44	-9.43
2	-21.53	-8.95	3.33	-7.15
3	-20.65	-10.61	1.11	-4.54
4	-20.48	-9.81	2.22	-7.83
5	-20.16	-11.06	3.39	-4.24
6	-19.54	-14.09	3.86	-2.97
7	-19.26	-12.46	10.09	-7.56
8	-19.10	-11.61	3.24	-5.57
9	-18.76	-11.80	2.55	-5.18
10	-17.44	-13.62	10.31	-4.34
**2H4Z-DBB**				
1	-23.46	-10.31	2.62	-6.75
2	-22.33	-10.82	0.81	-7.18
3	-20.85	-11.57	8.33	-7.69
4	-18.65	-10.71	3.33	-5.59
5	-18.52	-10.01	4.13	-5.81
6	-18.39	-10.40	4.65	-4.52
7	-17.95	-11.40	0.15	-1.30
8	-17.32	-9.96	1.01	-2.43
9	-17.24	-10.11	0.98	-4.89
10	-16.36	-6.71	2.06	-6.01
**Table 4.3**				
**2EEP-BZL**				
1	-36.74	-16.59	4.58	-11.17
2	-34.42	-14.40	2.68	-11.17
3	-31.79	-15.81	5.21	-8.95
4	-30.99	-13.96	3.70	-8.34
5	-30.85	-15.06	2.24	-7.08
6	-30.56	-14.39	3.85	-9.56
7	-30.24	-13.02	1.57	-8.33
8	-30.06	-12.17	0.41	-8.34
9	-29.29	-14.15	4.94	-9.42
10	-29.20	-11.20	2.50	-9.54
**2EEP-DMB**				
1	-33.30	-13.90	4.36	-10.59
2	-33.04	-14.99	3.22	-8.87
3	-31.69	-15.96	2.91	-8.50
4	-27.98	-16.70	17.54	-12.21
5	-27.86	-14.64	4.39	-7.72
6	-27.66	-14.57	4.62	-7.76
7	-27.41	-15.08	4.11	-6.69
8	-27.26	-15.93	2.53	-6.04
9	-26.86	-12.38	0.89	-8.36
10	-26.21	-11.82	2.20	-8.26
**2EEP-DFB**				
1	-31.74	-17.64	5.76	-6.61
2	-31.10	-17.47	7.28	-6.83
3	-31.09	-16.84	4.23	-6.74
4	-30.95	-14.87	4.37	-9.02
5	-29.59	-17.34	8.59	-6.64
6	-28.02	-15.47	5.12	-7.50
7	-27.33	-12.04	1.70	-7.48
8	-26.95	-12.05	2.34	-7.43
9	-26.87	-12.56	5.46	-9.25
10	-26.80	-14.20	1.98	-4.98
**2EEP-DCB**				
1	-36.83	-18.77	9.02	-10.91
2	-35.64	-16.44	3.86	-9.55
3	-35.31	-17.56	7.77	-9.57
4	-33.82	-15.25	3.39	-8.73
5	-32.32	-13.85	4.93	-10.42
6	-32.25	-13.36	1.05	-10.30
7	-31.96	-13.10	4.56	-10.43
8	-30.45	-15.06	3.75	-8.84
9	-27.90	-12.16	5.71	-10.31
10	-27.69	-15.23	4.91	-7.24
**2EEP-DBB**				
1	-32.51	-14.53	3.12	-8.32
2	-31.97	-14.38	2.22	-8.40
3	-31.83	-15.09	1.41	-7.99
4	-30.15	-15.36	6.11	-9.60
5	-28.43	-14.71	4.88	-7.93
6	-27.52	-13.74	2.83	-7.21
7	-26.42	-13.18	1.81	-7.16
8	-26.34	-12.74	1.54	-7.00
9	-25.86	-18.82	19.12	-8.12
10	-25.60	-15.40	3.15	-5.53

### 3.7 Quantitative structure and reactivity relationship studies (QSAR)

QSAR studies are the regression methods used in chemistry and pharmaceutical sciences, where we use a set of variables to predict the potency of the response variable. This model can be used to predict various physico-chemical properties of the molecules and relate them to the observed biological activities [44]. Binding energy = -101/-556/-336 (ionization energy) +53/-269/-101 (electron affinity) -493/-4305/-2433 (chemical softness) + 6/146/76 (electrophilicity index) +784/6717/3809 (3WMT/2H4Z/2EEP). The results indicate that for BZL to be better inhibitor of feruloyl esterase, bisphosphoglycerate phosphatase and prolylamino peptidase resulting in its inhibitor property, it has to have a more negative eletrophlicity index while the ionization energy, electron affinity (except for 3WMT) and chemical softness ought to be more positive (Table 5).

**Table 5:** Chemical descriptors and binding energies for QSAR study.

Compound	IP	EA	χ	μ	η	S	ω	BE(3WMT)	BE(2H4Z)	BE(2EEP)
BZL	8.748	6.065	7.407	-7.407	1.342	0.745	20.441	-8.89	-8.97	-11.17
DMB	8.689	5.880	7.285	-7.285	1.405	0.712	18.887	-7.12	-11.11	-10.59
DFB	8.681	5.702	7.192	-7.192	1.490	0.671	17.357	-6.05	-5.57	-6.61
DCB	8.174	5.708	6.941	-6.941	1.233	0.811	19.537	-8.31	-9.43	-10.91
DBB	8.694	5.905	7.300	-7.300	1.395	0.716	19.100	-6.81	-6.75	-8.32

### 3.8 IR and Raman spectra

Most of the simulated peaks are found to be in close agreement with the experimental peaks. Bromine compounds (Table 6) [45] shows strong vibrations in the region of 720–550 cm^−1^, due to the CBr stretch and bands at 670, 610 cm^−1^ (IR) and 664, 611 cm^−1^ (DFT) are assigned to this modes for DBB. For DCB, CCl stretches are noticed at 710, 690 cm^−1^ (IR) and at 719, 688 cm^−1^ (DFT) [46]. The υC-F is assigned at 1235, 1215 cm^−1^ (IR), 1218 cm^−1^ (Raman) and at 1227, 1225 cm^−1^ theoretically for DFB [47]. The υC = O [48] is usually found at 1750-1650 cm^−1^ and these are assigned at 1685, 1666 cm^−1^ (IR), 1675 cm^−1^ (Raman), 1671, 1669 cm^−1^ (DFT) for BZL, 1720, 1660 cm^−1^ (IR), 1660 cm^−1^ (Raman), 1667, 1665 cm^−1^ (DFT) for DMB, 1670, 1655 cm^−1^ (IR), 1672, 1658 cm^−1^ (Raman), 1668, 1666 cm^−1^ (DFT) for DFB, 1665 cm^−1^ (IR), 1670, 1667 cm^−1^ (DFT) for DCB and at 1670, 1650 cm^−1^ (IR), 1669, 1666 cm^−1^ (DFT) for DBB. For the title compounds, the ring breathing modes for para-substituted phenyl ring are assigned at 808 cm^−1^ (IR), 810, 788 cm^−1^ (Raman), 812, 787 cm^−1^ (DFT) for DMB, 805 cm^−1^ (IR), 808 cm^−1^ (Raman), 810, 806 cm^−1^ (DFT) for DFB, 835, 766 cm^−1^ (IR), 832, 769 cm^−1^ (DFT) for DCB and at 833, 763 cm^−1^ (IR), 829, 764 cm^−1^ (DFT) for DBB [49, 50]. The ring breathing mode of the mono substituted phenyl ring is assigned at 1013, 1006 cm^−1^ (IR), 1000 cm^−1^ (Raman) and at 1009, 1003 cm^−1^ (DFT) for BZL [51, 52, 53]. In order to investigate the performance and vibrational wavenumbers of the title compounds, root mean square deviation (RMSD) between calculated and observed wavenumbers were calculated [54]. The RMSD of the observed IR bands are found to be 5.1 (BZL), 10.3 (DMB), 7.8 (DFB), 4.7 (DCB) and 7.3 (DBB). The RMSD values are 3.3 for BZL, 4.6 for DMB and 5.1 for DFB.

**Table 6:** Vibrational assignments.

B3LYP/CC-pVDZ (5D, 7F)	IR		Raman		Assignments^a^
υ(cm^−1^)	IRI	RA	υ(cm^−1^)	υ(cm^−1^)	
**Table 6.1 BZL**					
3108	2.49	5.20	3106	-	υCH
3088	15.35	26.98	3088	-	υCH
3075	3.47	365.80	-	3075	υCH
3065	24.42	26.89	3062	-	υCH
3064	1.08	243.22	3062	3055	υCH
3053	0.35	19.28	3045	3048	υCH
1671	68.81	203.76	1685	1675	υC = O
1669	276.24	19.99	1666	-	υC = O
1584	12.46	31.63	1582	-	υRing
1457	0.67	16.09	1455	1456	υRing
1418	37.19	0.18	1405	-	υRing
1314	1.79	5.38	1320	1320	υRing
1286	9.23	18.54	1288	1288	δCH
1268	9.42	8.95	1266	-	δCH
1179	237.69	15.57	1185	1177	υCC
1061	9.53	0.02	1061	1057	δCH
1026	0.48	10.94	-	1025	δCH
1009	7.46	2.84	1012	-	υRing
1003	0.21	90.43	1000	1000	υRing
966	0.30	0.67	965	-	γCH
930	0.37	0.83	935	-	γCH
848	75.99	0.23	-	847	γCH
800	74.03	0.58	795	800	γCH
711	16.97	0.66	710	-	τRing
699	25.71	15.00	700	700	δRing
679	50.13	1.75	678	-	τRing
629	103.86	1.21	635	636	δRing
603	1.39	1.40	605	606	δRing
486	6.61	3.94	485	484	τRing
425	0.28	0.76	-	425	τRing
401	0.02	2.39	-	400	τRing
334	3.04	0.40	-	336	τRing
277	32.96	0.02	-	275	δRing
146	1.66	1.40	-	148	τRing
**Table 6.2 DMB**					
3107	0.85	13.43	3100	-	υCH
3085	0.01	149.69	3070	3070	υCH
3053	27.49	39.15	3050	3050	υCH
3046	31.23	1.27	3045	3030	υCH
3000	11.51	81.37	-	3000	υCH3
2998	15.50	71.18	-	2995	υCH3
2968	20.05	77.64	2965	2960	υCH3
2967	4.41	195.52	2965	2960	υCH3
2912	4.86	684.39	2910	2913	υCH3
2911	44.50	38.39	2910	2913	υCH3
1667	69.31	2.45	1720	-	υC = O
1665	290.83	24.15	1660	1660	υC = O
1593	227.58	93.33	1598	-	υRing
1592	32.38	713.12	-	1580	υRing
1549	2.40	3.84	1555	-	υRing
1411	40.78	2.71	-	1410	δCH3
1398	7.00	18.39	1400	-	δCH3
1379	10.23	0.08	1377	1377	δCH3
1340	0.01	83.01	1345	1340	δCH3
1339	0.23	11.38	1345	1340	δCH3
1304	4.91	0.02	1302	1300	υRing
1188	0.42	52.18	-	1190	δCH
1186	250.05	18.64	1184	1180	δCH
1143	245.11	10.23	1145	-	δCH
1089	18.74	1.26	1085	-	δCH
1024	0.13	9.29	1025	-	υCC
1009	4.09	0.70	-	1010	δCH3
992	10.35	0.06	998	-	δCH
957	0.65	7.13	955	-	δCH3
862	55.22	0.87	870	870	γCH
833	5.11	3.05	835	836	γCH
824	48.77	0.83	825	-	γCH
812	18.90	2.71	808	810	υRing
787	0.09	61.46	-	788	υRing
768	6.66	2.82	770	-	γCH
738	97.08	1.30	740	-	γCH
624	0.79	2.16	-	625	δRing
605	3.68	1.14	607	603	δRing
587	54.99	0.33	588	-	δRing
495	15.53	3.35	494	-	τRing
414	0.28	6.25	-	412	τRing
401	6.72	1.73	-	400	δRing
355	0.30	0.33	-	357	τRing
341	0.15	2/26	-	340	τCH3
249	12.12	1.04	-	250	τRing
248	19.01	1.50	-	244	τC = O
**Table 6.3 DFB**					
3116	2.65	7.17	-	3110	υCH
3095	0.18	278.20	3100	3095	υCH
3082	0.05	14.85	3083	-	υCH
1668	54.80	238.06	1670	1672	υC = O
1666	267.94	28.23	1655	1658	υC = O
1568	58.71	2.51	1555	-	υRing
1387	28.78	0.18	1382	-	υRing
1315	3.94	5.86	1320	1320	υRing
1312	10.57	0.91	1305	1306	υRing
1253	0.01	8.39	-	1250	δCH
1227	274.81	3.76	1235	-	υCF
1225	15.00	27.33	1215	1218	υCF
1177	285.78	15.20	1160	1165	δCH
1119	223.62	2.30	1110	-	δCH
1069	0.31	4.63	-	1060	δCH
1019	0.29	8.52	1020	-	υCC
970	0.01	0.42	-	972	γCH
945	4.12	0.56	957	-	γCH
841	99.96	1.68	845	850	γCH
840	64.44	2.64	-	840	γCH
810	1.63	56.71	-	808	υRing
806	4.83	7.99	805	-	υRing
758	36.09	0.71	757	760	τRing
691	5.56	0.38	-	700	τRing
619	4.80	2.26	-	622	δRing
603	1.02	0.86	600	600	δRing
501	1.49	0.09	500	500	τRing
433	1.40	3.81	-	435	τRing
415	19.67	0.28	-	420	δRing
407	0.09	0.30	-	405	τRing
356	1.07	2.29	-	355	τC = O
267	0.33	0.86	-	270	τC = O
261	26.65	0.65	-	258	τRing
226	0.04	1.25	-	225	τRing
**Table 6.4 DCB**					
3095	0.02	2.37	3100		υCH
3082	0.01	71.32	3080		υCH
3082	0.18	6.34	3080		υCH
1670	61.75	4.31	1665		υC = O
1667	304.59	2.95	1665		υC = O
1573	36.71	7.37	1575		υRing
1547	52.98	3.63	1535		υRing
1302	5.74	2.02	1308		υRing
1298	14.46	0.43	1297		υRing
1179	316.87	21.10	1177		δCH
1021	0.50	14.10	1015		υCC
971	0.17	0.52	965		γCH
948	0.07	1.06	946		γCH
862	54.58	0.12	858		γCH
832	34.15	3.88	835		υRing
769	46.83	0.28	766		υRing
733	0.45	35.82	735		γCH
719	16.68	1.11	710		υCCl
688	0.82	0.89	690		υCCl
476	3.13	0.92	470		τRing
**Table 6.5 DBB**					
3114	1.74	4.77	3100		υCH
3088	1.16	2.27	3090		υCH
3082	0.47	8.58	3050		υCH
1669	64.43	5.82	1670		υC = O
1666	318.06	3.70	1650		υC = O
1568	336.69	9.76	1570		υRing
1273	14.10	2.13	1275		υRing
1179	295.30	2.61	1177		δCH
1081	12.85	1.04	1080		δCH
1020	0.75	20.91	1015		υCC
970	0.20	0.53	965		γCH
947	0.04	1.34	945		γCH
829	26.95	4.49	833		υRing
764	50.50	0.24	763		υRing
724	7.13	2.01	725		γCH
664	14.08	6.31	670		υCBr
611	4.21	1.11	610		υCBr
496	28.90	1.28	498		δRing
467	4.82	0.70	465		τRing

a υ-stretching; δ-in-plane deformation; γ-out-of-plane deformation; τ-torsion;; IR_I_-IR intensity (KM/Mole); R_A_-Raman activity (Ǻ^4^/amu); Ring-Phenyl ring.

## 4 Conclusion

The present drugs were structurally, vibrationally, physico-chemically and biologically investigated using vibrational spectroscopy and theoretical tools. The scale simulated spectra and experimental IR and Raman spectra showed close agreement and all the vibrations were found to be in the expected range. LHE is maximum for DBB with value 0.9079. Photovoltaic modelling shows that DMB is the best compound to be used in the DSSC to get the best output. The value of BZL is almost same as that of DMB. The molecular charge configuration was displayed by which the orientation of electrophilic and nucleophilic zones was identified to recognize the storage of chemical potential in the molecular entities. The chemical reaction path for customizing chemical reactivity for the molecule was studied by chemical shift root among the core and allied carbons. The molecular property stabilization, driving potential and control mechanism inside the frontier interactive system was illustrated with the help of lobe degenerative system. Docking shows the ligands have good pharmacological properties with the proteins. From atomic contact energy and global energy values more stable complex are identified. QSAR predictions shows that BZL is a better inhibitor of feruloyl esterase, bisphosphoglycerate phosphatase and prolylamino peptidase resulting in its inhibitor property, it has to have a more negative eletrophlicity index while the ionization energy, electron affinity (except for 3WMT) and chemical softness ought to be more positive.

## Declarations

### Author contribution statement

Y. Shyma Mary, Y. Sheena Mary, K.S. Resmi, Veena S. Kumar, Renjith Thomas, B. Sureshkumar: Conceived and designed the calculations. Analyzed and interpreted the data; Contributed materials, analysis tools or data and software; Wrote the paper.

### Funding statement

This research did not receive any specific grant from funding agencies in the public, commercial, or not-for-profit sectors.

### Competing interest statement

The authors declare no conflict of interest.

### Additional information

No additional information is available for this paper.
